# Advancements in stem cells treatment of skeletal muscle wasting

**DOI:** 10.3389/fphys.2014.00048

**Published:** 2014-02-12

**Authors:** Mirella Meregalli, Andrea Farini, Clementina Sitzia, Yvan Torrente

**Affiliations:** Stem Cell Laboratory, Dipartimento di Fisiopatologia Medico-Chirurgica e dei Trapianti, Centro Dino Ferrari, Fondazione IRCCS Ca' Granda Ospedale Maggiore Policlinico, Università degli Studi di MilanoMilano, Italy

**Keywords:** muscle wasting, stem cell niche, fibrosis, inflammation, myogenic stem cell

## Abstract

Muscular dystrophies (MDs) are a heterogeneous group of inherited disorders, in which progressive muscle wasting and weakness is often associated with exhaustion of muscle regeneration potential. Although physiological properties of skeletal muscle tissue are now well known, no treatments are effective for these diseases. Muscle regeneration was attempted by means transplantation of myogenic cells (from myoblast to embryonic stem cells) and also by interfering with the malignant processes that originate in pathological tissues, such as uncontrolled fibrosis and inflammation. Taking into account the advances in the isolation of new subpopulation of stem cells and in the creation of artificial stem cell niches, we discuss how these emerging technologies offer great promises for therapeutic approaches to muscle diseases and muscle wasting associated with aging.

## Introduction

Skeletal muscle is a highly complex system formed by thousands of contractile units called muscle fibers. Each muscle fiber is limited by a plasma membrane called sarcolemma and by a basal lamina, that are surrounded by an extra cellular matrix constituted of connective tissue (Buckingham et al., 2003). Muscle remodeling occurs throughout the entire life although at different rate considering the developmental stages. Starting from embryo until childhood, protein synthesis is upregulated and satellite cells (SCs) actively develop new muscle fibers while in adults cellular turnover is strongly reduced (Schiaffino et al., 2007). In response to exogenous stimuli or to biological factors such as age or nutrition, the muscle increases its size, the amount of contractile proteins and consequently force production. The regulation of muscle cell size is a tightly regulated phenomena, and it is a balance between muscle proliferation and degradation of pre-existing proteins. Uncontrolled events, often associated with diseases, lead to hypertrophy or atrophy, respectively (Sandri, 2008). The complex hierarchy of events that triggers muscle remodeling is often unbalanced in muscular diseases. For instance, Duchenne muscular dystrophy (DMD), the most frequent among all the dystrophies, is characterized by a rapid atrophy in youth, muscular wasting and inability to walk in adolescence and premature death for cardiorespiratory failure by the age of 30. As the genetic nature of these pathologies leads to uncontrolled fiber degeneration, different treatments were proven to delay the progression of the diseases. The main goal was to retard the atrophy and replace diseased muscle with new healthy and functional muscle fibers by using myogenic stem cells (Brunelli and Rovere-Querini, 2008). Unfortunately, the use of stem cell in regenerative medicine is limited by the poor engraftment and persistence of transplanted cells and the risk of neoplastic formation (Suuronen et al., 2008; Kuraitis et al., 2012). Due to these findings, other aspects were deeply investigated to increase the survival of injected stem cells into pathological muscle. Modulation of the inflammatory reaction is a key step for stem cell transplantation (Smythe et al., 2000): myeloid cells (Suzuki et al., 1999; Mcnally et al., 2000), macrophages (Wehling et al., 2001; Villalta et al., 2009), neutrophils (Hodgetts et al., 2006) and eosinophils (Cai et al., 2000) actively contribute to development of pathogenesis in several myophaties but only macrophages and sometimes eosinophils play a role in muscle regeneration (Tidball and Villalta, 2010). Pathological conditions modify the microenvironment of stem cells (the so-called niche) preventing the activation of resident stem cells and reducing the success of exogenous cell therapies. Tissue engineering technologies may create a novel *in vitro* niche allowing the maintenance and propagation of SCs and enhancing their muscular potential.

In this review, we will describe the efforts that are necessary to design a successful therapeutic approach for muscular diseases, relating to find a functional stem cell population, to identify feasible matrix/polymer to engineer stem cells' niche and to modulate secondary—but relevant—effects of impaired muscle regeneration, as fibrosis and inflammation.

## Myogenic stem cells

### Embryonic stem cells (ESCs)

#### Introduction to ESCs

Embryonic stem cells (ESCs) are pluripotent cells derived from the early embryo that are characterized by the ability to proliferate over prolonged periods of culture remaining undifferentiated and maintaining a stable karyotype (Amit and Itskovitz-Eldor, 2002; Carpenter et al., 2003; Hoffman and Carpenter, 2005). ESCs differentiate into cells forming all 3 embryonic germ layers, and are characterized by self-renewal, immortality, and pluripotency (Strulovici et al., 2007). As ESCs possess the potential to differentiate into all normal tissues, the ability to derive and maintain these cells in culture opened the possibility to have an unlimited supply of differentiated cells to replace pathological tissues (Moon et al., 2006; Skottman et al., 2006).

#### Markers of ESCs

Cell origins are often defined by one or more cell-surface markers and intracellular epitopes unique to that particular cell type. hESCs are maintained in culture on feeder layers of heterologous cells and then differentiated into specific cell lineages (Takahashi and Yamanaka, 2006; Conrad et al., 2008). Stage-specific embryonic antigen citation(SSEA) markers are used to distinguish early stages of cell development and to denote pluripotency: hESCs express SSEA-3 and -4 during pluripotency and only SSEA-1 upon differentiation (Andrews et al., 1996; Thomson and Marshall, 1998; Thomson et al., 1998; Reubinoff et al., 2001). Nanog is a NK-2-type homeodomain gene encoding for a transcription factor that is critically involved in the self-renewal of stem cells. In 2005, Lin's group demonstrated that the tumor suppressor p53 binds to the promoter of Nanog, stimulating p53 (Lin et al., 2005). Octamer-binding transcription factor 4 (Oct-4) down-regulation is observed in differentiating cells (Rosner et al., 1990). It was suggested that only Oct-4 was necessary for the maintenance of pluripotency, but its expression level governed three cell fates once differentiation occurs. Similarly, Xu et al. published that the catalytic component of telomerase, telomerase reverse transcriptase (hTERT), was expressed in undifferentiated cells and down-regulated upon differentiation (Xu et al., 2001).

#### Limits of ESCs

Although the attentions that received, scientific and medical issues need to be addressed before hESCs can be considered safe for clinical applications (Leist et al., 2008). The American federal government severely restricted access and use of hESCs in 2001 but they were largely overturned by the Obama administration. Many organizations and countries have already banned reproductive cloning of human beings. As this procedure can be used to generate stem cells for therapeutic purposes, in countries where this type of cloning is legal, such as Australia and the United Kingdom, the created embryos must be destroyed within 14 days. Guidelines in using ESCs were proposed by the International Society of Stem Cell Research citation (http://www.isscr.org/guidelines/index.htm).

#### Myogenic potential of ESCs

Several lineages (blood, cardiac muscle and endothelial cells) were obtained by *in vitro* differentiation of ESCs, however for skeletal muscle several drawbacks arose, especially for the difficulty to identify a temporal expression of myogenic regulatory factors (Rohwedel et al., 1994). This way, in 2005 Bhagavati et al. co-cultured ESCs derived from normal mice with a preparation from mouse muscle enriched for myogenic stem and precursor cells. They transplanted ESCs into dystrophic mdx mice but unfortunately newly-formed muscle was occasionally seen (Bhagavati and Xu, 2005). Similarly, Barberi et al. described a stroma-free induction system to derive mesenchymal precursors and skeletal myoblast from hESCs. Following *in vitro* maturation, these cells were injected into tibialis anterior of immunodeficient scid mice and it was observed a long-term myoblast engraftment and the lack of teratomas (Barberi et al., 2007). As it was suggested that the lack of myogenic differentiation of ESCs was due to the impairment of myogenic signals in the mesoderm (Darabi et al., 2008a), Darabi et al. transiently expressed paired box 3 (Pax3) and paired box 7 (Pax7) during early mesoderm development and obtained several early embryonic skeletal myogenic progenitors (Darabi et al., 2008b, 2011). These cells were also implanted into mdx mice and gave rise to large numbers of skeletal muscle fibers and SCs, so that muscle force was ameliorated (Darabi et al., 2009, 2011). More recently, Sakurai et al. described that the elimination of bone morphogenetic protein 4 (BMP4) from serum-free ESC cultures together with the implementation of lithium chloride (LiCl) allowed the differentiation of these cells to myogenic progenitors cells. hESCs-derived progenitors showed a notable capacity of differentiation into skeletal muscle cells (Sakurai et al., 2009).

### Induced pluripotent stem cells (iPSCs)

#### Generation of iPSCs

Recent advances in the understanding of ESC biology included the identification of several master regulators of ESC pluripotency and differentiation (Takahashi and Yamanaka, 2006). Intensive study of ESC growth conditions has not yet produced a complete picture of the unique transcriptional and epigenetic state that is responsible for pluripotency and self-renewal in ESCs. Yamanaka's group identified four factors (Oct3/4, Klf4, Sox2, and c-myc) whose expression is sufficient to produce cells similar to ESCs, called induced pluripotent stem cells (iPSCs). The same factors were used to reprogram human fibroblasts to an ESC-like pluripotent state.

#### The new era of iPSCs

Now that embryonic tissue is no longer required to make a pluripotent cell, investigators have the ability to create tissue-based models of human disease based on cells derived from individual patients (Dimos et al., 2008; Park et al., 2008; Ebert et al., 2009; Soldner et al., 2009). Accordingly, iPSCs were efficiently used in murine models of sickle cells anemia and Parkinson's disease. Even if these cells were showed to be suitable for cell therapy, it has to be yet demonstrated the possibility to generate human iPSCs without introduction of DNA into the genome (to avoid oncogenic potential of undifferentiated iPSCs following the unsafe reintroduction of these genes), to ameliorate the efficiency of manipulation of human iPSCs and the capacity to obtain any desired cell types.

#### iPSCs and human disease

Since the work of Yamanaka was published, reprogramming of cells provided a realistic way not only to obtain lines from patients with incurable pathologies to investigate disease mechanisms and drug screening but to generate sufficient numbers of patient-specific pluripotent stem cells (Egawa et al., 2012). The generation of patient-specific iPSCs has the advantage of avoiding many of the ethical concerns associated with the use of embryonic or foetal material, and have no risk of immune rejection. Many cell types like motor neurons (Dimos et al., 2008), hepatocytes (Song et al., 2009), pancreatic insulin producing cells (Zhang et al., 2006), hematopoietic cells (Hanna et al., 2007), retinal cells (Carr et al., 2009), cardiomyocyte (Zwi et al., 2009) and mesenchymal stem cells (Lian et al., 2010), have been successfully derived from human iPSCs. Nelson et al. reported the use of iPSCs for myocardial repair in animal models of acute myocardial infarction (Nelson et al., 2009) while Ye used iPSCs in different hematological disorders (Ye et al., 2009).

#### Myogenic potential of iPSCs

As described above, to be used for clinical applications, iPSCs need to be generated in large amount in safety; this way, protocols to isolate and characterize these cells were largely improved. Mizuno et al. identified iPS-derived satellite-like cells by means the expression of the SM/C-2.6 antibody (Mizuno et al., 2010) while Darabi purified PDGFαR+Flk−1− murine iPS cells that expressed the myogenic factor Pax7 (Darabi et al., 2008a, 2011). In fact, the group of Perlingeiro recently isolated large quantity of Pax7+ human iPSCs (and ESCs) that, transplanted into dystrophic mice, engrafted well producing high amount of dystrophin and replenishing the satellite cell compartment (Darabi et al., 2012). Similarly, the expression of MyoD and Myf5 allowed the purification of myogenic iPS cells (Iacovino et al., 2011; Goudenege et al., 2012). Filareto et al. successfully obtained iPSCs from fibroblast of dystrophin/utrophin double knockout mice and engineered them with the micro-dystrophin gene. Injected into dystrophic mice, these cells engrafted well and improved muscle strength (Filareto et al., 2013). In parallel, Tedesco et al. generated mesoangioblast/mesenchymal-like cells from iPSCs of healthy and dystrophic patients: these cells were also modified to express constitutively the MyoD gene. Transplanted into model mice of LGMD-2A, iPSCs cells ameliorated their dystrophic phenotype (Tedesco et al., 2012).

### Satellite cells (SCs)

SCs are small progenitor cells originating from somites that lie between the basement membrane and sarcolemma of individual muscle fibers (Shi and Garry, 2006; Sambasivan and Tajbakhsh, 2007). SCs are normally present in healthy adult mammalian muscle as quiescent cells and are characterized by the expression of Pax7, that is fundamental for their maintenance and self-renewal, and by the absence of Myogenic differentiation 1 (MyoD) and myogenin, that conversely are necessary for myogenic differentiation. Once activated in response to specific stimuli such as oxidative stress, SCs up-regulate the expression of Myf5 to start their proliferation so that they differentiate into new myofibers, driven by specific factors such as MyoD, myogenin and myosin heavy chain (Whalen et al., 1990). Since the work of Montarras and colleagues (Montarras et al., 2005), different techniques for SCs isolation were assessed. Sacco et al. derived SCs from transplantation of one intact myofiber and demonstrated that once transplanted into dystrophic mice, SCs proliferated and contributed to form new muscle fibers (Sacco et al., 2008). Cerletti et al. isolated the skeletal muscle precursors (SMPs): injected into animal models, these SC-like cells restored dystrophin expression and, more importantly, were positioned into the SC niche, where they regulated the subsequent rounds of injury and repair (Cerletti et al., 2008). Similarly, the muscle side-population cells (mSP) isolated by Tanaka et al. engrafted into host SC niche, giving rise both to SCs and myonuclear population (Tanaka et al., 2009).

Autologous transplantation of genetically corrected SCs into patients suffering from muscular diseases could be our ideal approach (Price et al., 2007): unfortunately, it was demonstrated that the growth of SCs *in vitro* significantly reduced their *in vivo* myogenic potential, rendering their transplantation an inefficient technique (Tremblay et al., 1993; Mendell et al., 1995; Gussoni et al., 1997). To overcome these problems, several studies investigated the SC niches, as described in detail in section Satellite cells niche.

### Muscle-derived stem cells (MDSCs)

Besides SCs, muscle-derived stem cells (MDSCs) were isolated within the muscle, with the capacity of self-renewal and mesodermal differentiation. Sarig et al. identified a subpopulation of MyoD+ stem cells that formed muscle fibers but also osteogenic and adipogenic cells (Sarig et al., 2006). Tamaki et al. purified a subpopulation of CD34-CD45- cells that proliferated into myogenic, vasculogenic and neural cell lineages (Tamaki et al., 2007). Sca−1+CD34+ stem cells purified from murine muscle differentiated into myogenic and multimyeloid lineages *in vitro* and regenerated muscle *in vivo* (Torrente et al., 2001). Alessandri et al. showed that muscle-derived stem cells positive for desmin and vimentin differentiated *in vitro* into skeletal muscle fibers and neurons (Alessandri et al., 2004). Notably, Rouger et al. identified early myogenic progenitors that originated from SC niche, the MuStem cells; transplanted into Golden retriever muscular dystrophy (GRMD) dogs, these cells allowed the re-expression of dystrophin (Rouger et al., 2011).

### Mesenchymal stem cells (MSCs)

Mesenchymal stem cells (MSCs) are clonogenic and adherent cells, isolated from adult and foetal bone marrow and from other tissues and organs (Alhadlaq and Mao, 2004; Le Blanc and Pittenger, 2005; Beyer Nardi and Da Silva Meirelles, 2006): they are able to differentiate into several lineages (Zheng et al., 2007; Nesti et al., 2008). As MSCs were identified into muscle tissue biopsies, it was suggested that skeletal muscle could be an important source of MSCs for therapeutic interventions (Jackson et al., 2010). Transplanted into DMD patients, MSCs fused with host fibers and enhanced the activity of endogenous stem cells through the secretion of trophic factors (Ichim et al., 2010). Interestingly, De Bari et al. described the *in vitro* myogenic potential of MSCs isolated from adult human synovial membrane (De Bari et al., 2001). Following injection into dystrophic mice, these cells formed new myofibers, re-expressed the dystrophin and contributed to SCs replenishment (De Bari et al., 2003). Gang et al. showed that MSCs from umbilical cord blood differentiated into skeletal muscle, expressing late myogenic markers as MyoD (Gang et al., 2004). Riordan et al. described that hematopoietic precursors present in the bone marrow were protected from inflammatory damage by MSCs (Riordan et al., 2007) while Nemeth et al. demonstrated that MSCs can modulate the activity of macrophages and consequently inhibit inflammatory processes (Nemeth et al., 2009). The capacity of MSCs to modulate inflammation could be an important feature in the perspective of cell therapy in dystrophic patients as inflammation is a prominent component of the disease (as reviewed in detail in section Inflammation and repair mechanisms in skeletal muscle). Following these evidences, MSCs injection were proven to reduce inflammation in animal models for several human diseases, such as autoimmune arthritis and diabetes (Fiorina et al., 2009; Madec et al., 2009), multiple sclerosis (Constantin et al., 2009; Rafei et al., 2009a), lupus (Zhou et al., 2008), rheumatoid arthritis (Song et al., 2010) and autoimmune encephalomyelitis (Rafei et al., 2009b). Although all these encouraging results, several problems need to be solved. First of all, more efforts are needed to elucidate the origin of MSCs; moreover, protocols for isolation of the cells and their expansion *in vivo* have to be standardized.

### Muscle-derived CD133+ stem cells

Torrente et al. isolated stem cells from human normal and DMD biopsies expressing the glycoprotein CD133. CD133+ stem cells co-expressed CD34, CD45, and kinase insert domain receptor (KDR) and differentiated into muscle (Torrente et al., 2007). Moreover, Negroni et al. found that muscle-derived CD133+ stem cells co-expressed the satellite cell marker CD56 and eventually formed myosin heavy chain (MyHC)+ multinucleated myotubes (Negroni et al., 2009). As Phase I clinic trial demonstrated that infusion of these cells was safe and feasible (Torrente et al., 2007), muscle-derived dystrophic CD133+ stem cells were engineered to express a shorter but still functional dystrophin. Transplanted into dystrophic mice, CD133+ stem cells allowed the expression of dystrophin and the formation of new myofibers, improving murine muscular force. Interestingly, some of injected CD133+ stem cells were identified beneath the basal lamina, in SC-like position, thus expressing M-Cadherin (Benchaouir et al., 2007).

### Mesoangioblasts

Physically associated with the embryonic dorsal aorta in avian and mammalian species, mesoangioblasts are multipotent progenitors of mesodermal tissues, expressing α-smooth muscle actin (SMA) and retaining myogenic capacity (Tagliafico et al., 2004). Cossu et al. engineered these cells with human microdystrophin and demonstrated that they improved muscle function after injection into GRMDs (Sampaolesi et al., 2006; Cossu and Sampaolesi, 2007). In order to ameliorate their ability of migration, mesoangioblasts were exposed to Stromal cell-derived factor (SDF)-1 and tumor necrosis factor (TNF)-α so that, following transplantation into α-sarcoglycan KO mice, the large majority of α-sarcoglycan-expressing myofibers was reconstituted (Galvez et al., 2006). Similarly, Tedesco et al. transduced mdx-derived mesoangioblasts with a vector carrying the entire human dystrophin genetic locus. Injected into scid/mdx mice, these cells formed several muscle fibers expressing dystrophin and replenished the SC compartments (Tedesco et al., 2011). More recently, Cossu's group obtained mesoangioblasts from iPSCs of LGMD-2D patients that rescued the expression of α-sarcoglycans in dystrophic mice (Tedesco et al., 2012). According to these evidences, mesoangioblasts seemed to be feasible to treat MDs and they are currently being utilized in a phase I/II clinical trial (EudraCT no. 2011-000176-33).

## Artificial stem cell niche

### Satellite cells niche

SCs behavior is influenced by factors that are secreted by myofibers. SDF-1 can bind to receptor CXCR4 on the surface of SC activating a migratory response (Sherwood et al., 2004; Ratajczak et al., 2006) while M-cadherin enhance the adhesion of SC to myofibers allowing their fusion (Irintchev et al., 1994). Interestingly, SCs can regulate their own quiescence and self-renewal according to the expression of ligands for the Notch receptor family (Conboy and Rando, 2002; Conboy et al., 2003; Kuang et al., 2007). Like other stem cells, SCs can proliferate in a asymmetric manner, giving rise to one stem cell and one differentiated cell; and in a symmetric manner, originating two daughter cells retaining full stem cell potential (Morrison and Kimble, 2006). Asymmetric self-renewal is preferred in quiescient conditions while the other is typical in case of injury or disease. Each tissue-specific stem cell is located inside anatomically-defined microenvironment, called niche, surrounded by extracellular matrix (ECM) composed of a network of fibrillar proteins, growth factors, chemokines, cytokines and proteins that are present on the surface of neighboring cells. According to the interactions with these components, the cell choose self-renewal or a pathway of differentiation, following specific stimuli (Lutolf and Hubbell, 2005; Cosgrove et al., 2009). SCs reside in the niches that are positioned in a compartment between the myofiber plasma membrane and the basal lamina that surrounds the myofiber so that in the apical part of the niche they receive the signals from the myofibers while on their basal surface they are influenced by basal lamina signals (Collins et al., 2005; Kuang et al., 2008). SCs express several molecules to interact with the basal lamina and all its components (collagen, laminin, fibronectin) (Burkin and Kaufman, 1999). Conversely, the proteoglycan components of the basal lamina bind growth factors secreted by SCs such as basic Fibroblast Growth Factors (bFGF), and Insulin-like growth factor 1 (IGF-1) that regulate SC survival and proliferation (Golding et al., 2007; Le Grand et al., 2009). Other factors derived from cells that are not proximal to the niches or from the systemic circulation can influence SCs, such as myostatin, and wingless-type MMTV integration site family, member 3a (Wnt3a) (Mccroskery et al., 2003; Brack and Rando, 2007). These extrinsic factors play a fundamental role in aging, when the regenerative capacity of skeletal muscle declines (Grounds, 1987): for example, increased levels of circulating Wnt3a allowed the activation of β-catenin pathway in SCs, so that muscle regeneration is reduced and fibrosis is enhanced (Brack et al., 2008). The incredible complexity of niche regulation is the reason why, after removal from their *in vivo* localization, SCs—and other adult stem cells—rapidly lost their myogenic ability (Dykstra et al., 2006) so that they cannot be used in clinical trials (Farini et al., 2009). As Kuang and collaborators suggested, the balance among the signals deriving from the various components of the niche is necessary to maintain the myogenic potential of the SCs (Kuang et al., 2008).

Recent studies have focused on imitate the regulatory machinery of the *in vivo* SC niche, as a powerful tool to control stem cell function. Three dimensional (3-D) matrices are the model system that mimics the *in vivo* microenvironment, allowing the investigation of these physiologic events (Cukierman et al., 2001; Abbott, 2003). They can derive from cells or tissues while others can be composed of ECM proteins. Natural ECMs can be formed by various protein fibrils and fibers interwoven within a hydrated network of glycosaminoglycan chains, providing a structural scaffold. Fibrils, pores, elastin and collagen can be present and alter the biophysical properties of ECMs. Moreover, artificial synthetic materials were produced with similar structure. Polyethylene glycol (PEG)-based hydrogels were used for the maintenance of SCs *in vitro* (Lutolf and Hubbell, 2005) while, recently, Kloxin and colleagues developed PEG hydrogels that controlled matrix stiffness without toxicity to cells (Kloxin et al., 2009). As these matrices were able to alter biophysical properties in a non-invasive manner, they were used to investigate the progression of biophysical changes associated with muscle fibrosis or disease (Engler et al., 2004). Moreover, Lutolf et al. demonstrated that PEG hydrogels were suitable for single-stem cell clonal assays and resistant to non-specific cell adhesion mediated by protein adsorption (Lutolf et al., 2009a). However, further studies are necessary to define exactly all the components that constitute the microenvironment of the SCs and the molecular steps that regulate the transition between SCs quiescence and proliferation.

### Tem cell fate *in vitro*

*In vitro* stem-cell colture is carried out on flat coated with different substrates like collagen or laminin, on feeder-cell layers and within hydrogels synthetized from ECM components (for example collagen or Matrigel). Most frequently culture of stem cells was performed on rigid polystyrene tissue-culture plastic exposing cells to soluble factors in liquid media (Lutolf et al., 2009b).

These culture conditions are far from resemble the *in vivo* condition, where cells live in close proximity to each other and in contact with the ECM. Recently, 3D niche are still being explored and should be considered. Blau's group are studying the two-dimensional (2D) biomaterial culture systems deconstructing the niche and identifying and assessing the effects of individual niche components on stem-cell fate (Lutolf et al., 2009b). Normally, the effects of cell–cell interactions are studied by co-culturing; this strategy makes it difficult to discriminate the role of particular molecules.

*In vivo*, secreted growth factors and cytokines are mostly tethered to ECM components like proteoglycans. At the same time, receptor ligands are presented to stem cells surface and to nearby support cells. In both cases, molecule immobilization probably has the critical role of increasing protein stability, promoting persistent signaling and inducing receptor clustering (Irvine et al., 2002). A covalent binding of fibroblast growth factor 2 (FGF2) to a synthetic polymer stabilized the growth factor and increased its potency 100-fold relative to FGF2 in solution. Similarly, the epidermal growth factor (EGF) covalently tethered to a biomaterial scaffold, was shown to be more effective than its soluble counterpart in inducing mesenchymal stem cells differentiation and preventing Fas-ligand-induced death (Fan et al., 2007). Natural and synthetic matrices can be used to create cell-culture substrates with known elastic modulus providing diffusion of soluble molecules to the basal surface and the apical one, and can be used to test the relevance of homeostatic and disease related matrix stiffness to stem-cell behavior. Soluble factors in culture media used in combination with the tissue-culture matrix affect cell fate. Human MSCs expressed genes consistent with differentiation into distinct tissue-specific cell types when exposed to polyacrylamide gels with a range of stiffness typical of brain, muscle and bone (Engler et al., 2006). The effects of the physical properties of culture substrate on stem-cell fate are fully appreciated, culture platforms based on soft biomaterials are likely to replace, rigid, tissue-culture plastic. Within the niche, cells dialog with the surrounding ECM during development and in adulthood (Folkman and Moscona, 1978). Although some of these effects are probably due to alterations in the adhesive interactions and crosstalk between the ECM and the cell as they work to define each other, there is ample evidence suggesting that physical control of cell shape alone can act as a potent regulator of cell signaling and fate determination (Wozniak and Chen, 2009).

### Stem cell fate *in vivo*

Biomaterials technologies offer great opportunities to control the stem cell fate *in vivo*, especially in case of tissue damage. Two main modes of application have been proposed: one in which biomaterials are used as carriers for introducing stem cells into damaged, diseased or aged tissue, and one in which biomaterials are used to augment endogenous stem-cell function (Lutolf et al., 2009b). In regenerative medicine, stem cell transplantation has some limitations: survival and engraftment of transplanted stem cells and the disrupted biological environment characterized by abundant cell and tissue necrosis. Biomaterials have to be designed to act as carriers for local delivery of stem cells, supporting cells or molecular niche cues. Biomaterials may improve the effect of stem cell transplantation; they may be used as multifunctional stem-cell microenvironments. They have to increase the delivering and enhancing the viability of the cells, to function as support in order to increase the numbers of the cells and stimulate the function of endogenous stem cells. Moreover, biomaterials can deliver diffusible cytokines in order to promote the mobilization of endogenous cells involved in repair, to enhance survival and to stimulate self-renewal and expansion of the transplanted cells. Materials would enhance tissue regeneration, tissue function and overcome the adverse effects of disease or ageing (Conboy et al., 2005; Adams et al., 2007). Therefore, they could permit local and specific delivery of bioactive niche components able to inhibit and stimulate molecules and drugs that have to increase the number and the functions of transplanted stem cells. In order to obtain these benefits *in vivo*, materials have to be achieved by forming a scaffold that deliver biomolecules near the stem-cell niche or by targeted delivery of soluble microparticles or as carriers of such bioactive niche components (Adams et al., 2007). Recently, Rothenfluh et al. isolated polymer nanoparticles, sufficiently small to enter the matrix of the targeted tissues; then, they modified them with a biomolecular ligand for matrix binding. This way, the modified the matrix into a source of nanoparticles (Gu et al., 2008; Rothenfluh et al., 2008). Similarly, Gu and co-workers modified existing nanoparticles so that they were used for differential delivery and controlled release of drugs (Gu et al., 2008). Biomaterials aim is not only to create materials to control spatially and temporally the components of the niche but also to study microenvironmental regulation of stem cell proliferation and fate (Conboy et al., 2005). Artificial niches could incorporate appropriate “homing” signals that would attract endogenous stem cells and localize them by means of known cell—cell or cell—matrix adhesive interactions. Biomaterial research is focused on create artificial niche where cells could to be exposed to tethered signals that control stem-cell function and expansion by self-renewal division.

## Muscle pathophysiology

### Muscle fibrosis

Following injury, a cascade of events starts to repair damaged tissues. First of all, inflammatory cells phagocyte the cell debris and secrete growth factors and cytokines that allow the proliferation of other cell types in the site of injury, as described in details below (see section Inflammation and repair mechanisms in skeletal muscle). Then, SCs start to proliferate and differentiate, a process which ultimately ends with the formation of new muscle fibers. Unfortunately, in muscular pathologies, the deficiency of structural proteins leads to continuous cycles of myofiber degeneration and regeneration, so that the damaged muscle fibers cannot be replaced by new fibers, causing myofiber degeneration, inflammation and fibrosis (Grounds et al., 2005; Serrano and Munoz-Canoves, 2010). In particular, the inflammatory cells eliminate the basement membranes of necrotic fibers that cannot be used to build the new fibers: this condition leads to abnormal muscle fiber arrangement in dystrophic muscles. Due to the chronic persistence of inflammatory cells, dystrophic muscles are characterized by higher concentration of growth factors and cytokines, that induce the massive proliferation and activation of fibroblasts. Their activity causes the accumulation of fibrotic elements that are responsible for uncontrolled events such as remodeling of the basal lamina and formation of collagenous tissues (Serrano and Munoz-Canoves, 2010). Normally, the events of muscle regeneration are tightly controlled by the interplay among different molecules. Insulin-like growth factor (IGF) is a key element in controlling tissue activity: it binds to cell surface receptors and to IGF-binding proteins, exerting a fundamental role in modulating myofibroblast and SCs proliferation. The matrix metallo-proteases (MMPs) have the function to degrade the ECM and to recruit inflammatory and myogenic cells in the site of injury while Sca-1 inhibits myoblast proliferation, preserving the progenitor cells (Serrano and Munoz-Canoves, 2010). Transforming growth factor (TGF)-β is highly expressed in regenerating muscle and it is a key regulator of fibrosis' development (Zhou et al., 2006); often, it functions in synergy with connective tissue growth factor (CTGF), inducing fibrosis and promoting dedifferentiation of myoblasts (Vial et al., 2008). CTGF binds to IGF-binding proteins and it is associated with fibrotic remodeling.

In the case of MDs, especially in DMD, membranes lacking the members of the dystroglycan complex are vulnerable to mechanical and oxidative stress. Due to myofiber breakdown, myofibroblasts remained activated: these phenomena are associated with altered production of ECM components and the accumulation of these molecules that lead to muscle cell necrosis and fibrosis (Klingler et al., 2012). Fibrosis development was considered a progressive and irreversible pathologic phenomenon, but recent advances in knowledge of its development steps render this pathological feature amenable for clinical treatments. A better understanding of the factors that participate in fibrosis may help identify pharmacological targets capable of attenuating the progression of untreatable muscular diseases.

### Muscular hypertrophy and atrophy: two opposites of the same phenomenon

Skeletal muscle is the most abundant tissue in mammals and muscle remodeling occurs throughout the entire life. A fine regulated pathway determines the balance between new protein accumulation and degradation of pre-existing ones (Sandri, 2008). Different stimuli, originated by functional overload or aging, can modulate this pathway causing a shift in this balance toward one side. Besides of physiological conditions, this pathway is influenced by lots of inherited and acquired disorders such as MDs, cancer cachexia and commons drugs as glucocorticoids (Cassano et al., 2009). Among signals that can produce hypertrophy, IGF1 pathway is one of the best characterized. IGF-1Ec is expressed in response to mechanical stimuli and cellular damage and promotes both proliferation and differentiation of satellite cells, while in adult myofibers it increases DNA content per myofiber and can influence myosin phenotype (Bamman et al., 2001). The binding of IGF-1 to its receptor IGF1R, triggers the activation of several kinases including phosphatidylinositol-3-kinase (PI3K), the consequent production of PIP3 recruits protein kinase B (AKT). AKT plays a central role in muscle remodeling: it acts by either activating positive signal (mTor) or blocking negative pathway (Myostatin, apoptotic cascade, GSK3β). A trophy results from degradation of both myofiber number and protein contents, through calpain system, lysosomal and the ubiquitin-proteasoma pathways (Voisin et al., 1996; Lecker et al., 1999). Two genes were found up-regulated in atrophy models: muscle-specific ubiquitin ligase atrogin-1 (MAFbx) and muscle RING-finger protein-1 (MURF1); further studies showed that they were ubiquitin-ligase expressed only in skeletal and cardiac muscle (Bodine et al., 2001). Another important factor is nuclear factor kappa-light-chain-enhancer of activated B cells (NF-kB) which is involved in inflammatory pathway leading to TNF-α and INF-γ expression and it can induce the degradation of MyoD. Moreover, knock out of myostatin, a member of the TGF-β family, can lead to an enormous enlargement of skeletal muscle mass (Mcpherron et al., 1997). Myostatin is in fact the most important negative regulatory element of fiber synthesis and it is strictly regulated during myogenesis thanks to the presence of E-boxes, MEF2 and GRE binding sites (Spiller et al., 2002). In particular, myostatin is synthesized as a precursor, that is processed by furin proteases to generate a dimer composed by an N-terminal pro-peptide, bound to biologically active C-terminal fragment. When the pro-peptide is cleaved, myostatin is activated and interact with several proteins, such as follistatin. Interestingly, mice without the expression of this protein have a reduced body mass (Matzuk et al., 1995) while follistatin forced expression leads to muscular hyper-growth (Nakatani et al., 2008). To test whether lack of myostatin could ameliorate the symptoms of muscular diseases, Whittemore et al. demonstrated that in wild type mice the blocking of the protein increased muscle mass (Whittemore et al., 2003) while Bogdanovich et al. showed that this condition in mdx mice improved myofibers size and muscular force (Bogdanovich et al., 2002). According to these studies, Wagner et al. described a phase I/II clinical trial of MYO-029 (a neutralizing antibody to myostatin) in dystrophic patients. This trial did not demonstrate any improvement in muscle strength, but no side effects were assessed, except for hypersensitivity skin reactions. This trial was originally designed to test safety so that a bigger cohort of patient or different choice of samples are required to detect arrest of disease progression or minimal improvements in strength. Furthermore, results could be explained by the fact that the patients were selected at late stage of the disease when the regenerative response is exhausted and the myostatin substrate was eliminated (Wagner et al., 2008). Similar studies were conducted also with animal models of other muscular diseases but opposite results were obtained (Li et al., 2005; Ohsawa et al., 2006). Further works demonstrated that myostatin not only downregulates the expression of several myogenic genes (Amthor et al., 2002; Mcfarlane et al., 2008) but efficiently inhibits the proliferation of muscle progenitor cells (Thomas et al., 2000). The complexity of mechanism involving muscle growth and regeneration is further increased by the discovery of microRNA. Recently, skeletal muscle specific microRNAs able to interact with master regulatory genes in muscle development were found (O'rourke et al., 2007). As an example, miRNA206 can influence satellite cells behavior by modulating Pax3 and MET transforming gene (cMet) (Clop et al., 2006; Mccarthy et al., 2007).

### Inflammation and repair mechanisms in skeletal muscle

Injuries affecting skeletal muscle determine the activation of the immune system and activate a cascade of events that are required to clean cellular debris and to allow the replacement of lost fibers with new ones. Furthermore, immune cells promote regeneration through the release of growth factors (Brunelli and Rovere-Querini, 2008). After acute muscular damage neutrophils rapidly appear, followed by phagocytic macrophages which continue to increase in numbers until about 2 days post-injury. A second population of macrophages develops at about 4 days post-injury and it is characterized by a non-phagocytic phenotype (Tidball and Villalta, 2010). In parallel, myogenic precursors start to proliferate and differentiate by recapitulating developmental steps. Firstly, response to injury is mediated by Th1 cytokines (INFγ and TNFα) which trigger the activation of classic M1 pro-inflammatory macrophages (Gordon and Taylor, 2005). At a second stage, a population of M2 anti-inflammatory macrophages is predominant thanks to Th2 cytokines stimulation, such as interleukin (IL)−4, −10, −13. This phenotype-switch is required to stop inflammation and to permit the differentiation and fusion of satellite cells. This process is strictly regulated and several signals are known to be involved (Fadok et al., 2001; Arnold et al., 2007) but further studies are needed to better understand each phase. In MDs, skeletal muscles are subjected to chronic injuries that maintain a continue activation of the immune system. In fact, inflammatory infiltrates consisting of both macrophages and lymphocytes are present and elevated serum cytokines levels are detectable. Furthermore, a partial adaptive response to treatment with corticosteroid supports a role for the immune system in exacerbating muscular wasting (Backman and Henriksson, 1995). Progressive MDs like DMD are characterized by an initial phase that recapitulates the event observed in acute injury and repair. A second phase is dominated by chronic inflammation which triggers fibrosis deposition and atrophy. In fact in adult mdx mice a transition from M2a macrophages to M2c macrophages occurs in an attempt to control M1 cytolitic macrophages and to promote muscle regeneration through the release of IL-10 and IL-4 (Gordon, 2003; Horsley et al., 2003). M2 macrophages may also partecipate in activation of cytotoxic T-cells (which promote muscle damage through perforin-mediate process) and promote muscle fibrosis through arginase metabolism of arginine (Villalta et al., 2009; Tidball and Villalta, 2010).

The importance of modulating immune system cells was proven in different animal model of MDs, for example depletion of macrophages from mdx mice resulted in reduced muscle membrane lysis (Petrof et al., 1993). Furthermore, non-steroidal anti-inflammatory drug (NSAID) treatment was effective both in ameliorating muscle morphology and reducing macrophage infiltration (Serra et al., 2012) and anti-oxidant drugs (N-acetylcysteine) in mdx mice reduced necrosis by regulating TNF-α level (De Senzi Moraes Pinto et al., 2013). Recently an important role for acquired immunity in DMD pathogenesis has been pointed out by (Mendell et al., 1995; Hemmati et al., 2003; Flanigan et al., 2013) opening new perspectives in treatment of MDs.

## Conclusions

Skeletal muscle emerged as a promising tissue source for stem and progenitor cells that can be used in a variety of therapeutic applications. Skeletal muscle constitutes around one third of body weight in a healthy subjects (Gates et al., 2008). Muscle has an high capacity to repair itself after injury; this characteristic suggests that it serves as a reservoir for cells that participate in tissue regeneration processes (Usas and Huard, 2007). Several works described the ability of different muscle-derived stem cell populations to differentiate into multiple cell types, including osteoblasts, adipocytes, chondrocytes, myoblasts and endothelial cells. In addition, these cells showed regenerative, anti-inflammatory and anti-apoptotic properties. Each of these cell types is characterized primarily on the basis of their *in vitro* characteristics after they have been isolated from the body. *In vivo* they exhibited the capacity to migrate through different tissues where they are exposed to different extracellular and environmental signals. While rudimentary models were developed to describe the *in vivo* relationship among these stem cell populations, substantial additional studies are needed to refine and verify these relationships.

New approaches using organisms genetically modified and transgenic mouse models proposed the importance of the microenvironment—like the niche and the extrinsic factors—to be a key component in stem cell regulation. Particularly, significant progress has been made in understanding how satellite cells can act as tissue-specific adult stem cells in skeletal muscle. In the same time, many studies investigated the satellite cell properties in term of efficacy after *in vivo* transplantation using novel approaches such as non-invasive bioluminescence imaging. These tools provided information for assessing not only satellite cell function but, in general, stem cell function. Investigations on the molecular nature of stem cell niche signals on *in vivo* models and short-term cultures of isolated myofibers, are now on-going. Bioengineering offers significant tools for the development of strategies to mimic biochemical and biophysical features of the *in vivo* niche microenvironment (Lutolf et al., 2009b). We hope that the synthesis of biomaterials, micro-fabrication technology and stem cell biology will provide systems potentially innovative to better understand how stem cell fate is controlled. The analysis of the niche and the dynamic responses of stem cells to well-defined artificial microenvironments, might give us the possibility to understand the role of specific niche components and niche architecture in regulating fundamental cellular mechanisms such as cellular division, self-renewal, and differentiation *in vitro* and *in vivo*. Development of biomaterials able to re-create an *in vitro* SCs niche could give rise to novel insights into understanding the molecular cues, critical for the *in vitro* maintenance and expansion of muscle stem cells. Above all, these *in vitro* systems can well lead to the generation of adequate numbers of stem cells and the ability to control their differentiation in order to maximize their utility, not only as cell-based therapeutics for tissue regeneration and replacement, but also as the control of inflammation after muscle damage (Cosgrove et al., 2009). In conclusion, all these considerations will be important not only to better characterize satellite cell biology and therapeutic approaches to treat muscle diseases and aging-related muscle wasting, but also to give necessary information for the study of adult tissue-specific stem cells.

## Author contributions

Mirella Meregalli and Yvan Torrente designed the approach, Andrea Farini and Clementina Sitzia wrote the manuscript.

### Conflict of interest statement

The authors declare that the research was conducted in the absence of any commercial or financial relationships that could be construed as a potential conflict of interest.
